# Increased IFN-γ-producing Th17/Th1 cells and their association with lung function and current smoking status in patients with chronic obstructive pulmonary disease

**DOI:** 10.1186/s12890-019-0899-2

**Published:** 2019-07-26

**Authors:** Weihan Xu, Ruimin Li, Yongchang Sun

**Affiliations:** 1 Department 2 of Respiratory Medicine, Beijing Children’s Hospital, Capital Medical University, National Center for Children’s Health, Beijing, China; 2 Department of Respiratory Medicine, Beijing Tongren Hospital, Capital Medical University, Beijing, China; 3 Department of Respiratory Medicine, Beijing Daxing Teaching Hospital, Capital Medical University, Beijing, China; 4 Department of Respiratory and Critical Care Medicine, Peking University Third Hospital, Beijing, China

**Keywords:** Lymphocyte, CD4^+^ T cell, Inflammation, COPD, Cytokines, Th17 plasticity

## Abstract

**Background:**

Th17 cells are believed to be important proinflammatory cells in the pathogenesis of chronic obstructive pulmonary disease (COPD). Recent evidence demonstrates that Th17 cells display substantial developmental plasticity, giving rise to Th17/Th1 cells that secret both IL-17 and IFN-γ and are more pathogenic in inflammatory diseases. The aim of this study was to examine the distribution of circulating Th17/Th1 subpopulation and its association with disease severity in patients with COPD.

**Methods:**

Blood samples were obtained from 21 never-smokers, 31 smokers with normal lung function and 83 patients with COPD. The frequencies of Th17 cells and the Th17/Th1 subset were measured using flow cytometry. Plasma concentrations of IL-6, transforming growth factor (TGF)-β1 and IL-12 were determined by ELISA. The associations of Th17/Th1 cells with lung function and smoking were evaluated.

**Results:**

In peripheral blood, significantly increased proportions of Th17/Th1 cells among CD4 cells and Th17 cells were found in COPD patients compared with never-smokers and smokers with normal lung function. The percentages of Th17/Th1 cells showed correlations with forced expiratory volume in 1 (FEV_1_) % predicted value (*r* = − 0.244, *p* < 0.05), and higher proportions of Th17/Th1 cells in GOLD stage IV patients compared with stage I patients. The percentages of Th17/Th1 cells were significantly higher in current smokers compared with ex-smoker COPD patients, and positively correlated with pack-years of smoking (*r* = 0.352, *p* < 0.01). The plasma concentrations of IL-6, TGF-β1 and IL-12 were significantly increased in patients with COPD compared with never-smokers and smokers with normal lung function.

**Conclusion:**

Our results revealed correlations of proportions of IFN-γ-producing Th17/Th1 cells with lung function and smoking, suggesting that increased Th17/Th1 cells may play a role in COPD progression.

## Background

Chronic obstructive pulmonary disease (COPD) is characterized by persistent airflow limitation and an enhanced chronic inflammatory response to noxious particles or gases, particularly cigarette smoke [1, 2]. Evidence shows that chronic inflammation, present in the peripheral and central airways, lung parenchyma and the systemic circulation, contributes significantly to the development and progression of COPD [3–5].

Inflammation in COPD was believed to be driven by T helper 1 (Th1) response, but accumulating evidence supports a critical role of Th17 response in the disease. Increased Th17 cells were found in the bronchial submucosa, airway epithelium, lung tissue, bronchoalveolar lavage and peripheral blood from COPD patients compared with smokers without COPD and healthy subjects [4, 6–8]. Inverse correlations were observed between Th17 cells and forced expiratory volume in 1 s (FEV_1_) percentage predicted in COPD [8]. IL-17 orchestrates the recruitment of neutrophils and macrophages by enhancing production of a variety of chemokines, such as IL-1β, IL-6, TNF-α, CXCL8, granulocyte colony-stimulating factor (G-CSF), and GM-CSF from inflammatory and structural cells of the lung [9, 10]. Moreover, a recent study found that IL-17A contributed to cigarette smoke-induced lymphoid neogenesis of late-stage COPD, suggesting that IL-17A is critical in chronic inflammation and adaptive immune responses in COPD [11]. However, IL-17A expression is not sufficient to define the pathogenic activity of Th17 cells, which represent heterogeneous populations with distinct trafficking profiles and abilities to provoke autoimmune diseases [12].

Recent studies identified a subset of IL-17/IFN-γ double-positive T cells, namely Th17/Th1cells, in inflamed tissues or blood from both humans and mice with chronic inflammatory disorders [13]. Th17 cells have been found to exhibit high plasticity because they convert to Th17/Th1 cells under inflammatory environments, especially IL-12-rich microenvironment, whereas Th1 cells cannot convert to Th17 cells [14, 15]. Th17/Th1 cells coexpress both Th1 and Th17 transcription factors consisting of T-box expressed in T cells (T-bet) and RAR-related orphan receptor (ROR)γt, and differ from Th1 cells on CD161, IL-17 receptor E and CCR6 expressions [13, 15]. Th17/Th1 cells display pro-inflammatory characteristics by higher expression of genes encoding cytokines, chemokines and transcription factors, such as *Cxcl3, Ccl3, Ccl4, Ccl5, Il22, Il3, Icos, Tbx21* and *Stat4*, and downregulated expression of genes encoding cytokines associated with immunoregulation, such as Il9, Il10, Ahr and Maf (which encode molecules involved in the regulation if IL-10 production), and therefore can be more pathogenic and aggressive [16]. Recent studies revealed the clinical relevance of Th17/Th1 cells in patients with autoimmune diseases and diabetes [17–19]. Moreover, Th17/Th1 cells are resistant to glucocorticoid-mediated T cell suppression, which may be of clinical implication in inflammation unresponsive to glucocorticoids, such as COPD.

Therefore, we examined the distribution of this novel pro-inflammatory Th17/Th1 cells in the peripheral blood of patients with COPD and smokers by using flow cytometry and correlated the frequency of Th17/Th1 subset with Global Initiative for Chronic Obstructive Lung Disease (GOLD) classification, lung function parameters and smoking status.

## Methods

### Subjects

The study population was recruited in Beijing Tongren Hospital and Beijing Daxing Teaching Hospital, Capital Medical University, China. There were 83 stable COPD patients, all current or former smokers, and 31 smokers and 21 never-smokers with normal lung function. Peripheral blood was obtained from all participants after written informed consent. The study was approved by the local research ethical committee (TRECKT 2008–14).

The diagnosis of COPD was established according to the criteria by GOLD guidelines [2]. COPD patients had an impaired pulmonary function (post-bronchodilator FEV_1_/forced vital capacity < 70%) and a smoking history of ≥20 pack-years. All participants with COPD were stable and had no exacerbations for ≥3 months prior to recruitment. Individuals with upper respiratory tract infection in the past 4 weeks, restrictive lung diseases, other chronic systemic inflammatory diseases, such as rheumatoid arthritis (RA), inflammatory bowel disease, were excluded. Some patients were treated with inhaled bronchodilators and inhaled corticosteroids (ICS), but did not receive oral steroid therapy. Individuals with a smoking history of ≥20 pack-years and post-bronchodilator FEV_1_/ FVC > 70% and FEV_1_ > 80% predicted value were categorized as smokers with normal lung function, including current and ex-smokers. Ex-smokers were defined as those quitting smoking for a minimum of 2 years before entering the study.

### Cell collection and flow cytometry analysis

Peripheral blood samples were drawn in ethylenediaminete-traacetic acid tubes from all participants and separated to peripheral blood mononuclear cells (PBMC) by centrifugation on Ficoll-Paque Plus solution (Amersham Biosciences, Amersham, Bucks, UK), at 400×g for 20 min at 21 °C. Then, PBMCs were washed by divalent cation-free Hanks balanced salt solution at 300×g for 5 min at 4 °C and resuspended at 10^6^ cells/ml in RPMI-1640 medium.

For cytokine analysis, freshly processed human PBMCs were stimulated with 50 ng/ml of phorbol 12-myristate 13-acetate and 500 ng/ml of ionomycin and incubated at 37 °C in the presence of 5 μg/ml Brefeldin A. After 5 h cells were collected and stained as previously demonstrated with anti-hCD4-PE (BD Biosciences, San jose, California, USA) for 30 min at room temperature. For detection of intracellular cytokines, cells were subsequently stained with anti-hIL-17-FITC (eBioscience, San Diego, California), anti-hIFN-γ-FITC (eBioscience) after fixation and permeabilization. Cells were analyzed by FACS-Calibur (BD Biosciences) and isotype control was used to set gates. A total of 1 × 10^6^ events were examined for each subject. The data were presented using proportions of cells and were analyzed by FlowJo software (Tree Star, Ashland, OR, USA).

### Cytokine enzyme-linked immunosorbent assay

The concentrations of IL-6, TGF-β1 and IL-12 in the plasma from the participants were measured by enzyme-linked immunosorbent assay (ELISA, eBioscience, San Diego, CA, USA) according to the manufacturer’s recommendations with the sensitivity of 2 pg/ml, 8.6 pg/ml, and 0.5 pg/ml, respectively.

### Statistical analysis

Parametric data were depicted as a mean and SD or as median and IQR when appropriate. For data not distributed normally, across-group comparison of three groups was made using the nonparametric Kruskal-Wallis test. When the test detected statistical significance, post hoc analysis for comparison between two groups was performed by the Mann-Whitney test. Correlations were analyzed by Spearman’s rank correlation coefficients.

## Results

### Demographic and clinical characteristics of the study population

The demographic and clinical characteristics of the patients with COPD, smokers and never-smokers with normal lung function were summarized in Table 1. There was no statistical difference in age and sex ratio among the groups. The lung function parameters were significantly different among the groups (all *p* < 0.001). There was no difference in smoking history of smokers and COPD patients. There were 17 current smoker and 29 ex-smoker COPD patients who were using ICS, including 20 GOLD I-II and 26 GOLD III-IV patients.

**Table 1:** The demographic and clinical characteristics of all participants

No. of Subjects	Never-smokers	Smokers	Patients with COPD
	*n* = 21	*n* = 31	*n* = 83
Age (years)	64.9 ± 6.5	63.7 ± 8.9	67.0 ± 8.4
Male/Female	19/0	29/2	79/4
Current/ex-smokers	0/0	27/4	36/47
smoking, pack-yrs	0	40 (23–78)	48 (31–109)
FEV_1_% predicted	93.5 ± 8.2	90.5 ± 6.4	49.3 ± 10.7
FEV_1_/FVC%	78.4 ± 5.1	72.9 ± 6.0	51.6 ± 9.3
Inhaled corticosteroid use	0	0	46

Values are presented median (IQR) for smoking history, mean and standard deviation for age, FEV_1_% predicted and FEV_1_/FVC%, n for all others. *COPD* chronic obstructive pulmonary disease, *FEV*_*1*_ forced expiratory volume in 1 s, *FVC* forced vital capacity

### Frequencies of circulating subpopulations of total CD4^+^ T cells in patients with COPD, smokers and never-smokers

**Fig. 1:**
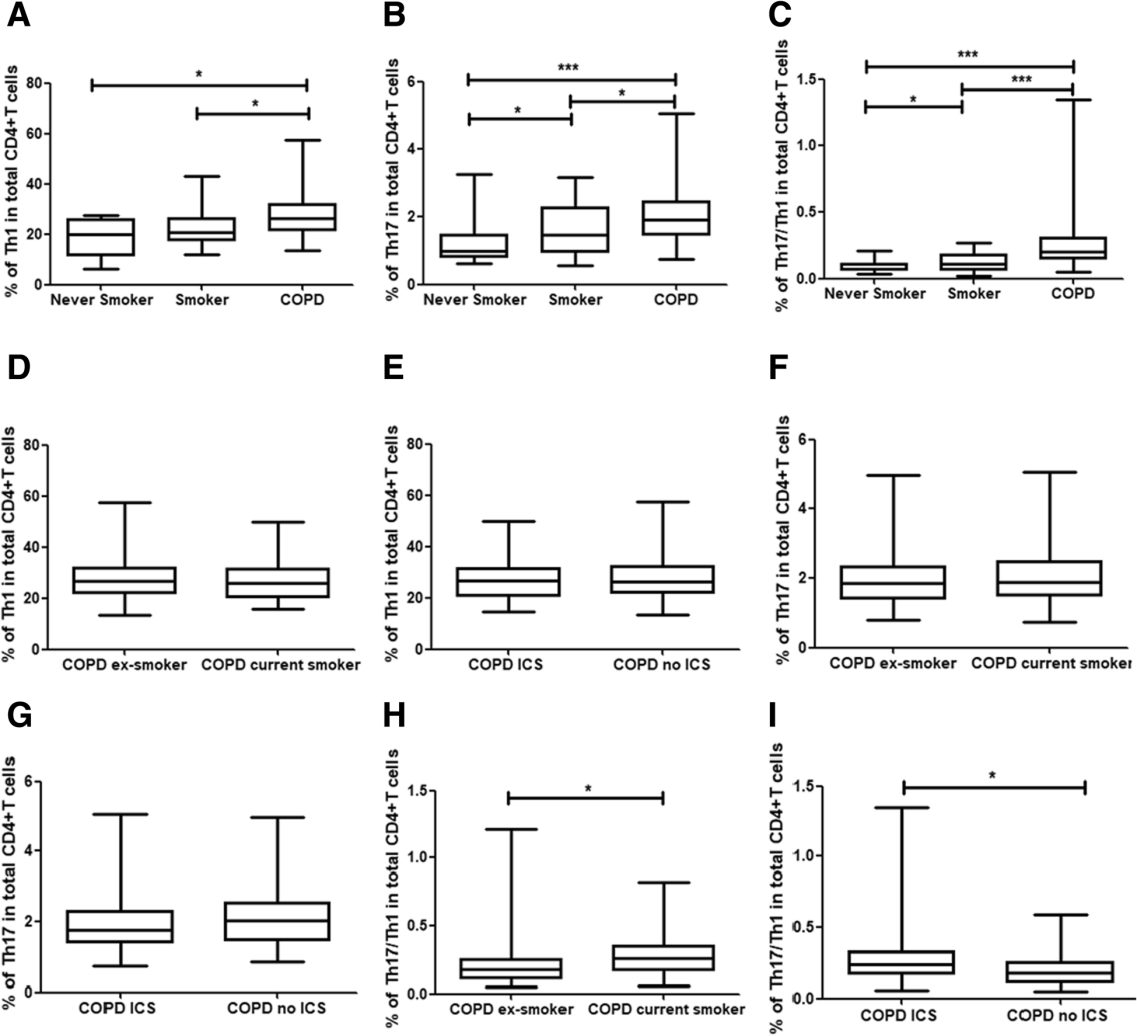
CD4+ T cell subsets in peripheral blood of the study populations. Three subsets of CD4+ T cells defined by the expression of interferon-γ and interleukin-17. **a**-**c** Frequencies of three subsets of CD4+ T cells in current smokers and ex-smoker COPD patients (**d**, **f**, **h**). Frequencies of three subsets of CD4+ T cells in COPD patients with and without ICS use (**e**, **g**, **i**). The boxes represent interquartile range while whisker displays the range. A *p* value< 0.05 was considered statistically significant

**Fig. 2:**
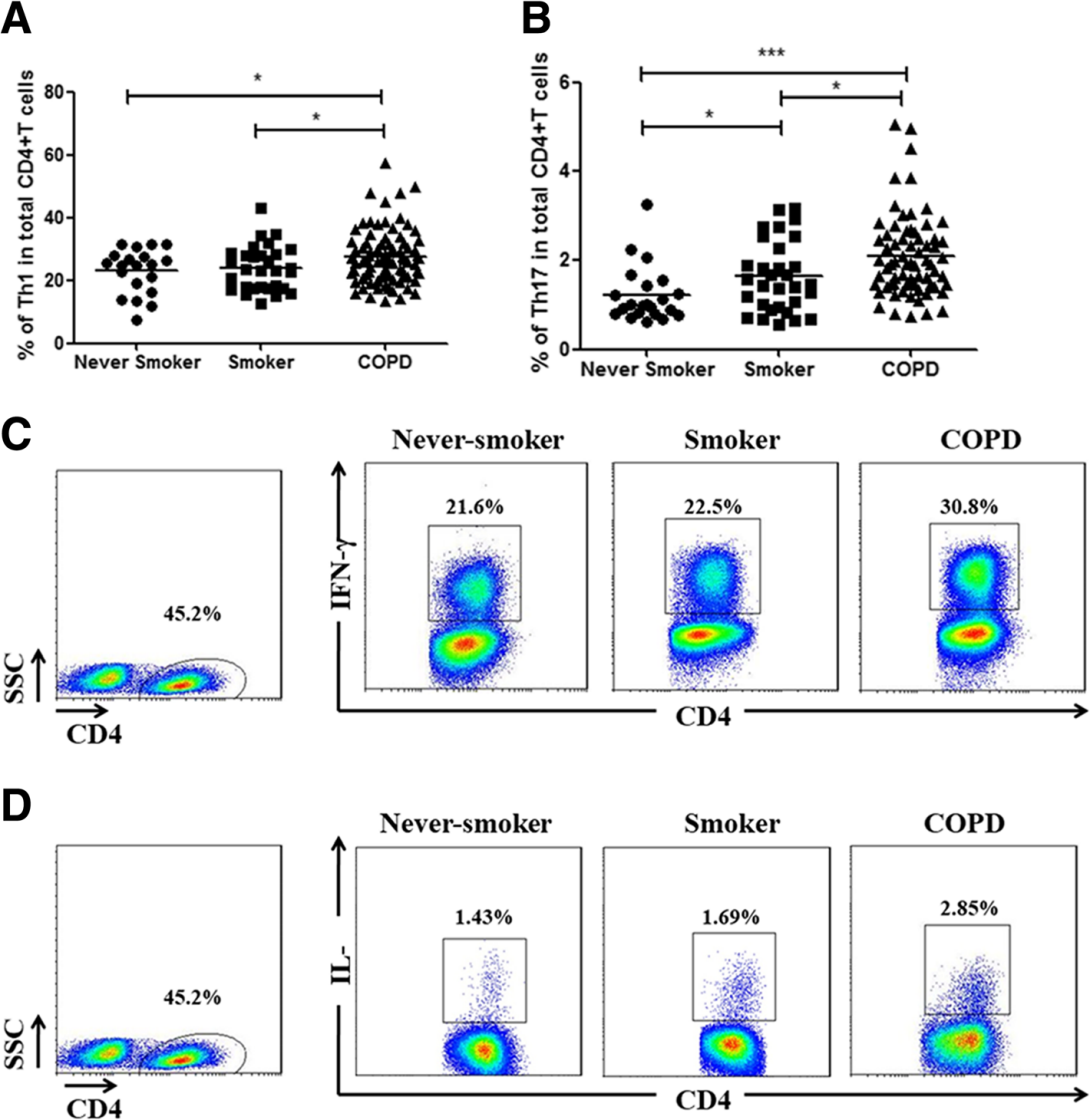
CD4^+^ T cell subsets in peripheral blood under physiological and disease conditions. Three subsets of CD4^+^ T cells defined by the expression of interferon-γ and interleukin-17. **a**-**b** Representative flow cytometry of Th1 and Th17 cells were shown. **c**-**d** The proportions of subsets among CD4^+^ T cells in never-smokers and smokers with normal lung function, and patients with COPD. Data were presented as median (IQR). Horizontal lines indicate median values. A *p* value< 0.05 was considered statistically significant

We first investigated the cytokine profiles of IFN-γ and IL-17 in CD4 + T cells in peripheral blood from the study participants using flow cytometry. Proportions of Th1 cells were significantly increased in patients with COPD (median 26.30%) compared with never-smokers (median 19.75%, *p* < 0.001) and smokers (median 20.60%, *p* < 0.01), and there was a trend for increase in smokers compared with never-smokers (Fig. 1a, Fig. 2a and c).

Patients with COPD showed significantly increased frequencies of Th17 cells in total CD4^+^ T cells (median 1.90%), in comparison to never-smokers (median 0.99%, *p* < 0.05) and smokers (median 1.46%, *p* < 0.01). And there was a higher percentage of Th17 cells in smokers than in never-smokers (*p* < 0.05) (Fig. 1b, Fig. 2b and d).

### Detection of dual-positive Th17/Th1 cells in peripheral blood from patients with COPD, smokers and never-smokers

**Fig. 3:**
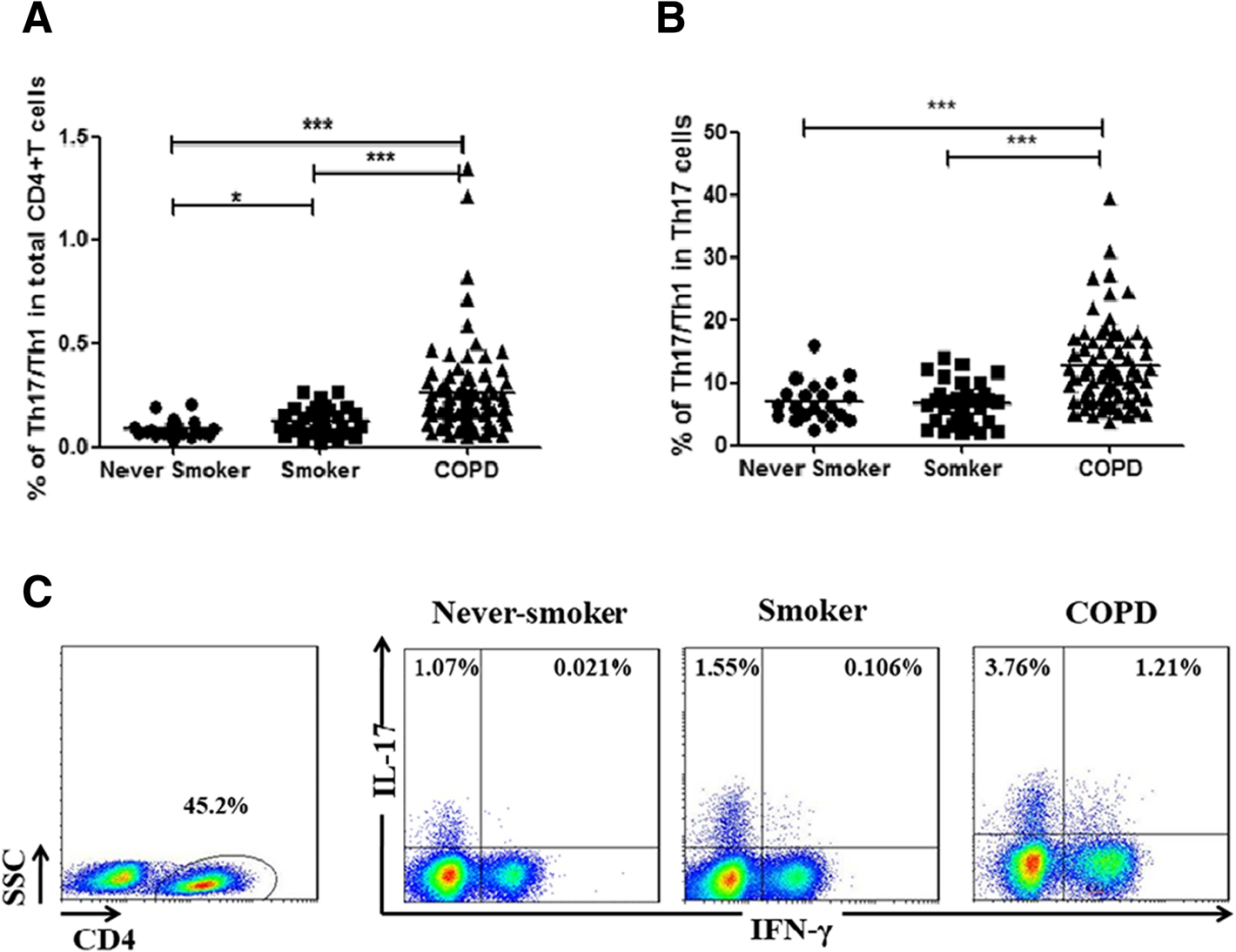
Expression of dual-positive Th17/Th1cells in peripheral blood from never-smokers and smokers with normal lung function, and patients with COPD. The CD4 + T cell population was analyzed for production of interferon-γ and interleukin-17. **a**-**b** Frequencies of Th17/Th1 cells among CD4^+^ T cells and Th17 cells in never-smokers, smokers and patients with COPD. **c** Representative flow cytometry of Th17/Th1 cells were shown. Data were presented a median (IQR). Horizontal lines indicate median values. A *p* value< 0.05 was considered statistically significant

In patients with COPD, the proportions of Th17/Th1 cells, identified as CD4^+^IFN-γ^+^IL-17^+^, among CD4 cells (median 0.203%) in the peripheral blood were significantly higher compared with never-smokers (median 0.075%, *p* < 0.001) and smokers (median 0.107%, *p* < 0.001) (Fig. 1c and Fig. 3a). Furthermore, significantly higher frequencies of Th17/Th1 cells among Th17 cells were observed in COPD patients (median 11.01%) compared with never-smokers (median 6.14%, *p* < 0.001) and smokers (median 7.15%, *p* < 0.001), suggesting increased differentiation of Th17 cells to Th17/Th1 cells in COPD (Fig. 3b). There also was a higher percentage of Th17/Th1 cells in smokers than in never-smokers (*p* < 0.05) (Fig. 3a and b). Moreover, the frequencies of Th17/Th1 cells among total CD4^+^ T cells in COPD patients using ICS (median 0.238%) were significantly higher compared with COPD patients not using ICS (median 0.176%, *p* < 0.05; Fig. 1i).

### Correlations between frequencies of Th17/Th1 cells and FEV_1_% in patients with COPD

**Fig. 4:**
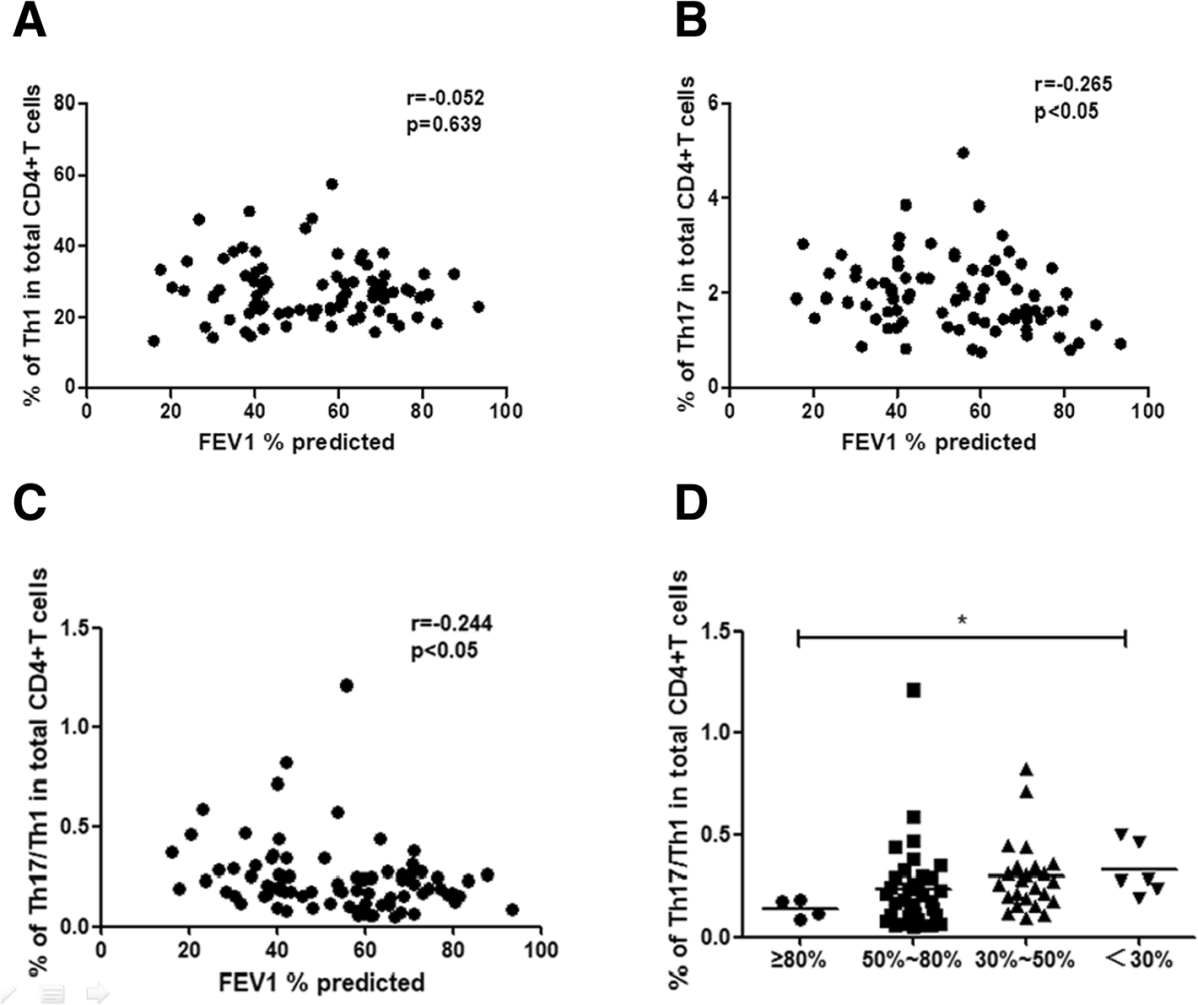
Correlations between frequency of Th17/Th1 cells with lung function in patients with COPD. Correlations between frequency of (**a**) Th1 cells, **b** Th17 cells, **c** Th17/Th1 cells with forced expiratory volume in 1 (FEV_1_) % predicted value. **d** Proportion of Th17/Th1 cells in COPD patients with different Global Initiative for Chronic Obstructive Lung Disease (GOLD) stages. A *p* value< 0.05 was considered statistically significant

To evaluate a possible correlation with disease severity, we analyzed the distribution of CD4^+^ T cell subsets within the COPD population with different FEV_1_% predicted values. There were no correlation observed between the percentages of Th1 cells and FEV_1_% predicted values (*r* = − 0.052, *p* = 0.639; Fig. 4a). There was a negative correlation between the percentages of circulating Th17 cells and FEV_1_% predicted values (*r* = − 0.265, *p* < 0.05; Fig. 4b). Moreover, increased percentages of circulating Th17/Th1 cells in COPD patients were inversely correlated with FEV_1_% predicted values (*r* = − 0.244, *p* < 0.05; Fig. 4c). Patients in GOLD stage IV had higher proportions of Th17/Th1 cells when compared with stage I patients (*p* < 0.05; Fig. 4d).

### Effects of smoking on frequencies of Th17/Th1 cells in patients with COPD

**Fig. 5:**
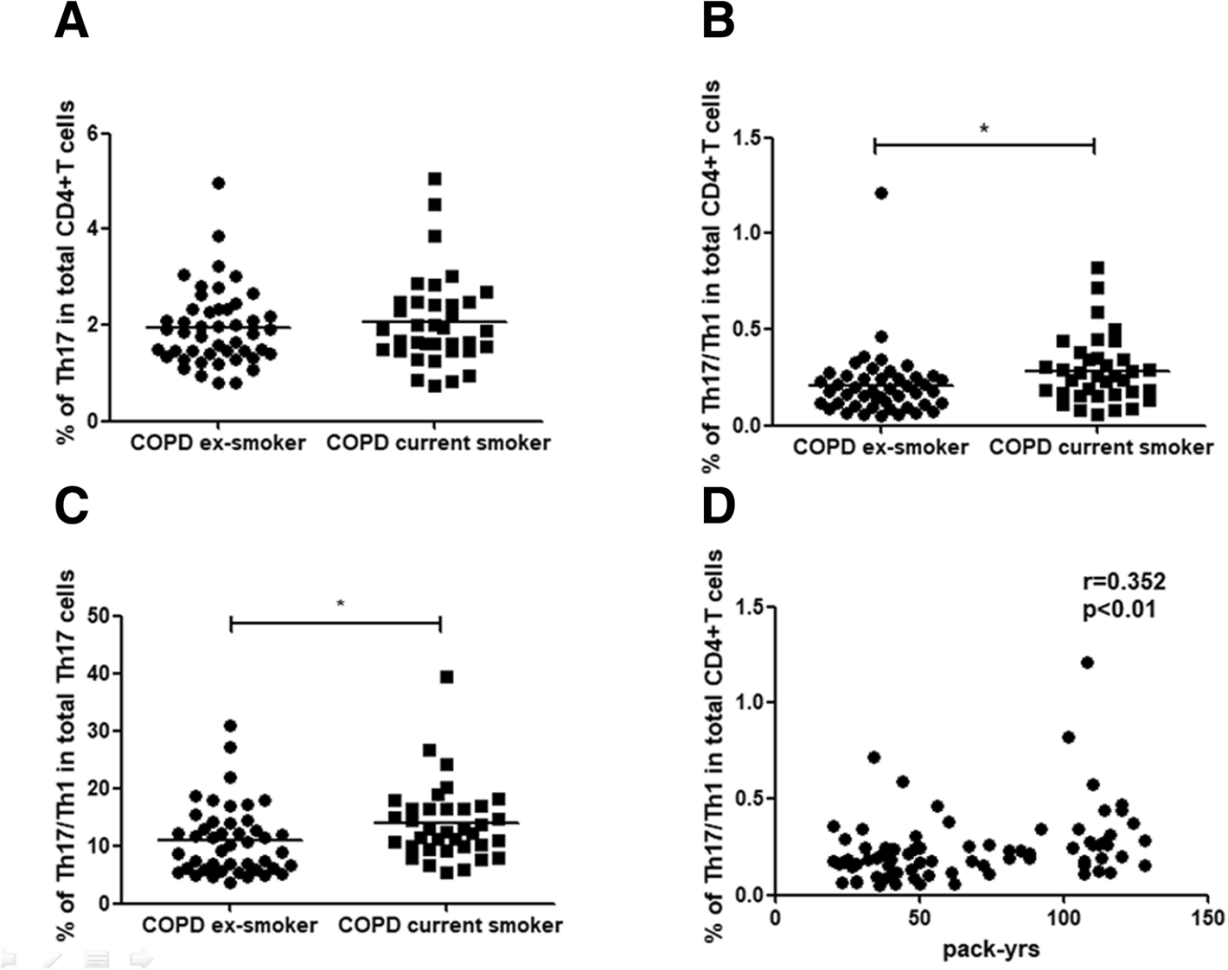
Effect of current smoking status on proportions of Th17 and Th17/Th1 cells in patients with COPD. **a**-**c** Frequencies of Th17 cells and Th17/Th1 cells in current smokers and ex-smoker COPD patients. **d** Correlations between frequency of Th17/Th1 cells with pack-years of smoking. Data were presented a median (IQR). Horizontal lines indicate median values. A *p* value< 0.05 was considered statistically significant

To assess the potential impact of current smoking on Th17/Th1 cells, we examined the differences in Th17/Th1 cells between current and ex-smoker COPD populations. We found no differences in the percentages of Th17 cells between current and ex-smoker COPD patients (*p* < 0.05; Fig. 5a). Interestingly, the frequencies of Th17/Th1 cells among total CD4^+^ T cells in current smoker COPD patients (median 0.261%) were significantly higher compared with ex-smoker COPD patients (median 0.177%, *p* < 0.05; Fig. 5b). Furthermore, current smoker COPD patients had higher proportions of Th17/Th1 cells among Th17 cells (median 12.46%) compared with ex-smoker COPD patients, suggesting a potential effect of cigarette smoke exposure on differentiation of Th17 cells to Th17/Th1 cells in COPD (median 10.09%, *p* < 0.05; Fig. 5c). In addition, there was a positive correlation between the percentages of circulating Th17/Th1 cells and pack-years of smoking (*r* = 0.352, *p* < 0.01; Fig. 5d).

### Plasma concentrations of IL-6, TGF-β1 and IL-12 in patients with COPD, smokers and never-smokers

**Fig. 6:**
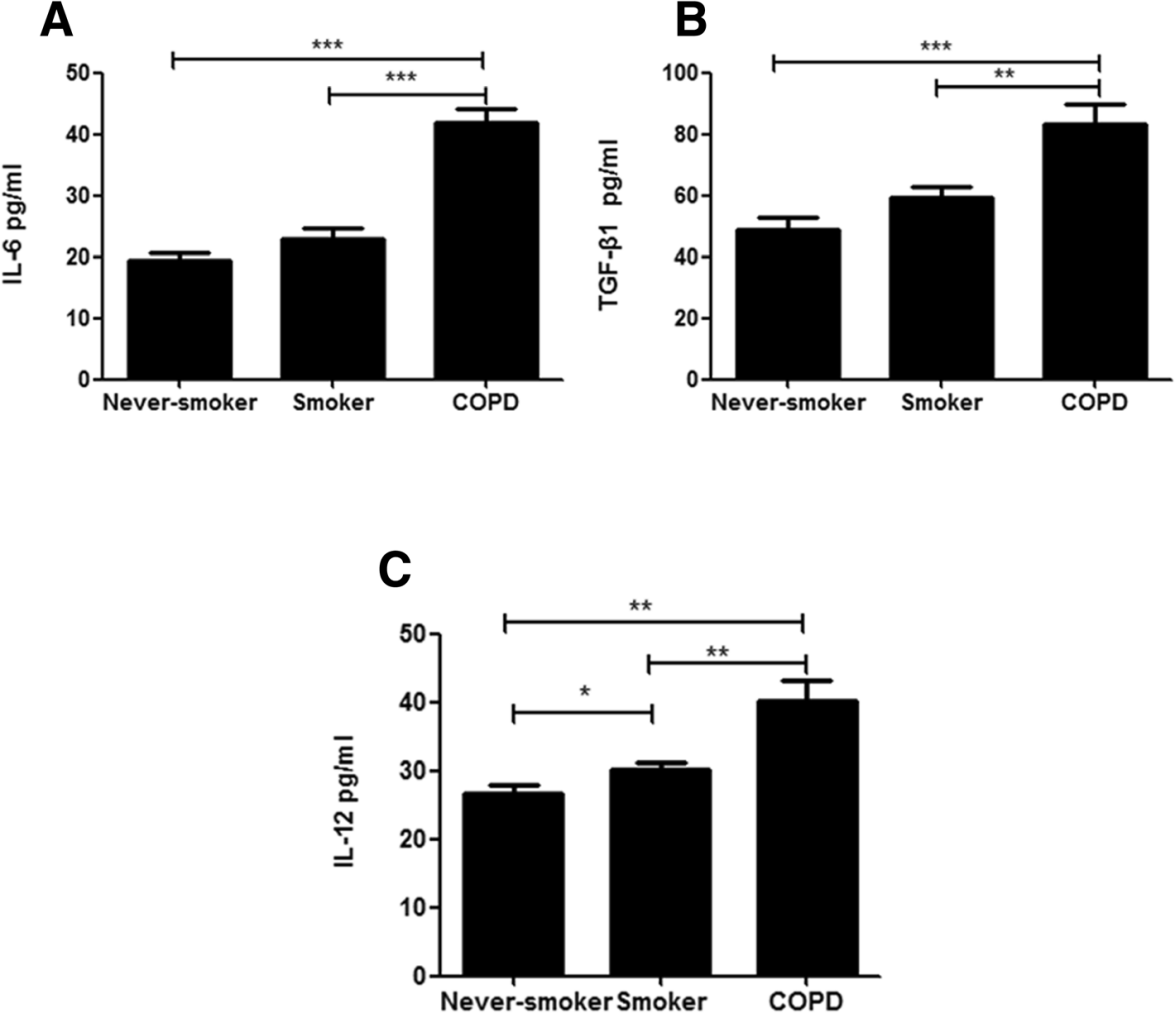
The concentrations of plasma cytokines. **a** Interleukin-6, **b** transforming growth factor-β1, and (**c**) Interleukin-12 from never-smokers and smokers with normal lung function, and patients with COPD. A *p* value< 0.05 was considered statistically significant

We examined the concentrations of plasma cytokines believed to drive Th17 cell differentiation and further conversion to Th17/Th1 cells. In plasma from patients with COPD patients, the concentrations of IL-6, TGF-β1 and IL-12 were significantly higher compared with never-smokers (*p* < 0.001, *p* < 0.001, *p* < 0.01, respectively) and smokers (*p* < 0.001, *p* < 0.01, *p* < 0.01, respectively) (Fig. 6a-c).

## Discussions

Th17 cells are a recently identified CD4^+^ T subset with pro-inflammatory actions, and are associated with human autoimmune diseases. IL-17A secretion is not sufficient to define the pathogenic activity of Th17 cells, and not all Th17 cells are pathogenic [12]. Function and phenotypic heterogeneity of human Th17 is a considerable barrier for understanding their contribution in diseases. Emerging data have identified IFN-γ and IL-17 dual-positive Th17/Th1 cells as potentially pathogenic Th17 cells [12, 14, 16–22]. A study by Cosmi et al found that a shifting from Th17 cells to Th17/Th1 cells occurred in synovial fluid of juvenile idiopathic arthritis patients, and the frequencies of Th17/Th1 cells were higher and positively correlated with parameters of inflammation [17]. Harbour et al showed that transition of Th17 cells to Th1-like cells was required for pathogenesis of colitis [18]. In lymphopenic mice, type 1 insulin-dependent diabetes was induced by Th17 cells only after their conversion into Th1 cells, and the onset of the disease was prevented by anti- IFN-γ, but not anti-IL-17 neutralizing antibody [19].

As COPD is a lung disease with significant systemic inflammation, we hypothesized that conversion of Th17 cells to Th17/Th1 cells may occur in the periphery and associate with disease manifestations. Here, we found, for the first time to our knowledge, that percentages of Th17/Th1 cells from COPD patients not only increased among CD4 cells, but also among Th17 cells, compared with smokers and never-smokers with normal lung function. These data suggest that the increased proportion of Th17/Th1 cells among CD4^+^ T cells was not only due to increased number of Th17 cells, but also due to increased late differentiation of Th17 cells to Th17/Th1 cells. More importantly, we revealed a negative correlation between the frequency of circulating Th17/Th1 cells and FEV_1_% predicted, suggesting a role of these cells in COPD pathogenesis.

Another interesting finding emerging from our study was the demonstration that the percentages of Th17/Th1 cells among CD4 + T cells, as well as among Th17 cells, were significantly higher in current smoker COPD patients than in ex-smoker COPD patients, and positively correlated with pack-years of smoking, although there was no difference in the percentages of Th17 cells among COPD smokers and ex-smokers. A study by Ammitzbøll et al found that GPR15 + T cells were associated with a Th17/Th1 phenotype and correlated with disease activity in multiple sclerosis smokers [23]. Smoking was shown to induce the expression and methylation of GPR15, and methylation of GPR15 was linked to the cumulative exposure to smoking and could be reversed by smoking cessation [24, 25]. Recently, Bauer et al found that tobacco smoking induced an excess in the GPR15-expressing T cells subsets [26]. It is conceivable that conversion of Th17 cells to Th17/Th1 cells in COPD may be driven by smoking exposure via induction of GPR15, which warrants further investigation.

Since Th17 plasticity is driven by inflammatory conditions [14], we supposed that the increased Th17 plasticity was related to the systemic inflammation of COPD. We found significantly higher levels of IL-12 in the plasma from COPD patients compared with smokers and healthy controls, suggesting that the circulating microenvironment in COPD may also contribute to the late plasticity of Th17 cells to Th17/Th1 cells.

Our study had several limitations. We investigated only peripheral blood, not bronchoalveolar lavage or lung tissue specimens, which may be more relevant to the pathogenesis of COPD. In addition, some of the COPD patients had used ICS, and therefore the possibility of an effect of ICS on the results cannot be excluded, although studies found no correlations between ICS and T cells in COPD, except for IL-17F^+^CD4^+^ T cells [4]. In our study COPD patients using ICS had a higher frequency of Th17/Th1 cells, this maybe due to the higher proportion of GOLD III and IV patients who were taking this medicine, considering the negative correlation between the frequency of Th17/Th1 cells and FEV_1_%predicted.

## Conclusions

In summary, our study provided new data for understanding the potential role of a new subset of Th17/Th1 cells in COPD. These findings may facilitate our understanding of the underlying mechanisms for systemic inflammation and disease progression in COPD.

## Data Availability

The datasets used and/or analyzed during the current study available from the corresponding author on reasonable requests.

## Abbreviations

COPD: Chronic obstructive pulmonary disease
ELISA: Enzyme-linked immunosorbent assay
FEV_1_: Forced expiratory volume in 1 s
FVC: Forced vital capacity
GOLD: Global Initiative for Chronic Obstructive Lung Disease
ICS: Inhaled corticosteroid
IFN-γ: Interferon-γ
IL-17: Interleukin-17
PBMC: Peripheral blood mononuclear cells
Th1: T helper 1
TNF-β1: Transforming growth factor-β1
